# Author Correction: The long-term effects of chemotherapy on normal blood cells

**DOI:** 10.1038/s41588-025-02315-x

**Published:** 2025-07-31

**Authors:** Emily Mitchell, My H. Pham, Anna Clay, Rashesh Sanghvi, Nicholas Williams, Sandra Pietsch, Joanne I. Hsu, Nina Friesgaard Øbro, Hyunchul Jung, Aditi Vedi, Sarah Moody, Jingwei Wang, Daniel Leonganmornlert, Michael Spencer Chapman, Ellie Dunstone, Anna Santarsieri, Alex Cagan, Heather E. Machado, E. Joanna Baxter, George Follows, Daniel J. Hodson, Ultan McDermott, Gary J. Doherty, Inigo Martincorena, Laura Humphreys, Krishnaa Mahbubani, Kourosh Saeb Parsy, Koichi Takahashi, Margaret A. Goodell, David Kent, Elisa Laurenti, Peter J. Campbell, Raheleh Rahbari, Jyoti Nangalia, Michael R. Stratton

**Affiliations:** 1 Wellcome Sanger Institute, Hinxton, UK; 2 Wellcome–MRC Cambridge Stem Cell Institute, Cambridge Biomedical Campus, Cambridge, UK; 3 Department of Haematology, University of Cambridge, Cambridge, UK; 4 Signalling Programme, The Babraham Institute, Babraham Research Campus, Cambridge, UK; 5 Department of Molecular and Cellular Biology, Baylor College of Medicine, Houston, TX USA; 6 Department of Clinical Immunology, Copenhagen University Hospital, Rigshospitalet, Copenhagen, Denmark; 7 Department of Paediatrics, Cambridge University Hospitals Foundation Trust, Cambridge, UK; 8 Department of Oncology, Cambridge University Hospitals Foundation Trust, Cambridge, UK; 9 Oncology R&D, AstraZeneca, Cambridge, UK; 10 Department of Surgery, University of Cambridge, Cambridge, UK; 11 Cambridge Biorepository for Translational Medicine, NIHR Cambridge Biomedical Research Centre, University of Cambridge, Cambridge, UK; 12 Department of Leukemia, Division of Cancer Medicine, MD Anderson Cancer Center, University of Texas, Houston, TX USA; 13 York Biomedical Research Institute, Department of Biology, University of York, York, UK

**Keywords:** Cancer, Genomics

Correction to: *Nature Genetics* 10.1038/s41588-025-02234-x, published online 1 July 2025.

In the version of the article initially published, Nina Friesgaard Øbro was missing from the author list and contributions. The information has now been added to the HTML and PDF versions of the article.

