# Supplementary material for: The long-term effects of chemotherapy on normal blood cells
Source: Nat Genet. 2025 Jul 1;57(7):1684–94. doi: 10.1038/s41588-025-02234-x (PMC12283364; doi:10.1038/s41588-025-02234-x)
Supplement: Supplementary file 1 — Supplementary Methods, Figs. 1–4 and refs. 1–11. [file 41588_2025_2234_MOESM1_ESM.pdf]

nature genetics

Supplementary information https://doi.org/10.1038/s41588-025-02234-x

The long-term effects of chemotherapy on normal blood cells

In the format provided by the authors and unedited

Supplementary information

Table of Contents

Supplementary Methods **2**

Supplementary Figure 1: Flow sorting strategy for single HSPCs *2*

Single-base-substitution and indel calling *2*

Insertion/ deletion calling *3*

Structural variant and copy-number calling *3*

Supplementary Figure 2: Validation of variant filtering approach *3*

Construction of phylogenetic trees *4*

Analysis of driver variants *5*

Bulk cell sorts for Nanoseq sequencing (chemotherapy-exposed samples) *6*

Supplementary figure 3: Flow sorting strategy for bulk cell sorts (chemotherapy exposed) *6*

Supplementary Figure 4: Flow sorting strategy for bulk cell sorts (unexposed) *7*

Nanoseq (duplex) sequencing *8*

HDP mutational signature analysis *8*

References **10**

Supplementary Methods

Supplementary Figure 1: Flow sorting strategy for single HSPCs

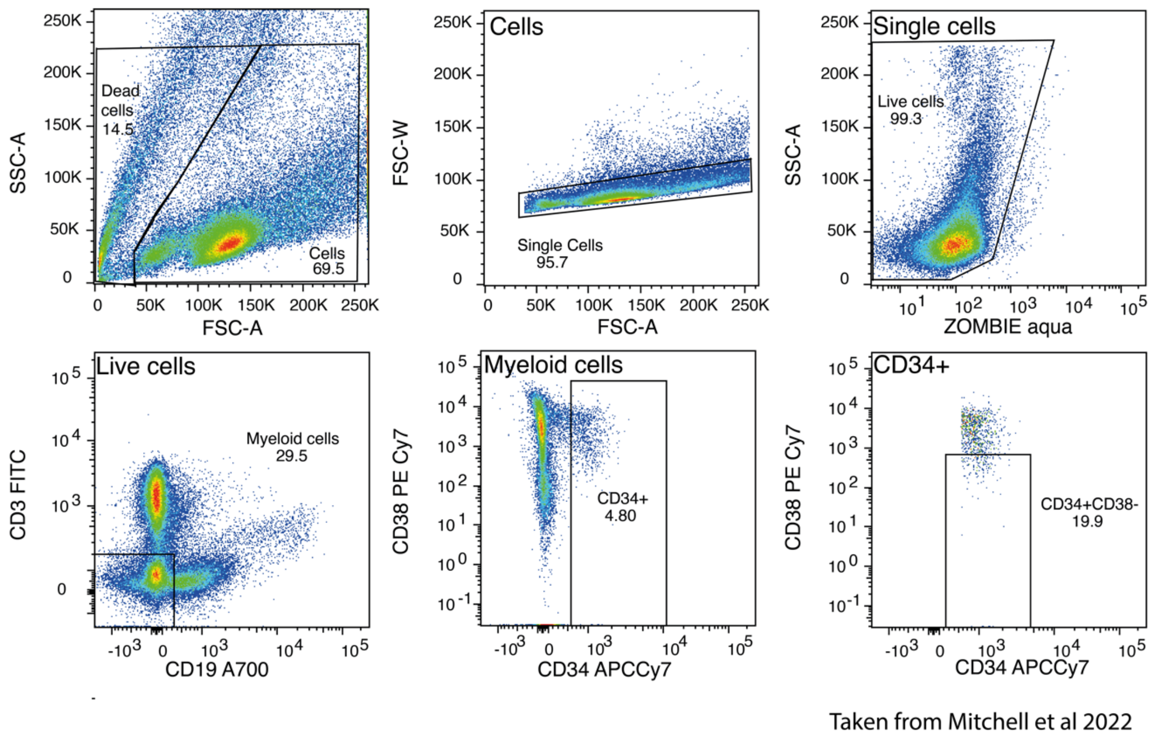

Supplementary Figure 1| Flow-sorting strategy for single HSPCs (CD34+CD38-) cells. A, Sorting of single human HSPCs from cord blood, peripheral blood and bone marrow. Cells were stained with the panel of antibodies in **Table S3** then single HSPCs were index sorted according to the strategy depicted into individual wells of 96 well plates.

Single-base-substitution and indel calling

The method for substitution calling involves three main steps: mutation discovery, filtering, and genotyping, as described in a previous paper[1].

Mutation discovery is initiated using the CaVEMan algorithm[2], configured with copy-number settings of major copy number 5 and minor copy number 2 for normal clones to maximize sensitivity. An unmatched normal sample is utilized to prevent the misclassification of embryonic mutations as germline mutations.

Various filters are applied to the data. Filtering against a panel of 75 unmatched normal samples helps eliminate common single-nucleotide polymorphisms (SNPs). Additional filters target mapping artefacts associated with BWA-MEM alignment, setting thresholds such as requiring a median alignment score ≥140 and less than half of reads to be clipped. Fragment-based statistics are employed to prevent calling variants supported by a low number of fragments. Variants are further annotated and filtered based on fragment coverage, number of fragments supporting the variant, fragment-based allele fraction.

Stringent criteria, including a variant allele frequency (VAF) >0.2, depth >7, and ≥4 mutant reads, are employed for mutation calling. The genotyping stage involves creating a pile-up of all samples from an individual, counting mutant and wild-type reads (*cgpVAF*; https://github.com/cancerit). Positions with insufficient data or conflicting information across samples are marked as not applicable (NA) for tree construction. Germline positions are identified based on consistent presence or NA status across samples from an individual.

Insertion/ deletion calling

Indels were called with the Pindel algorithm[6] using a matched normal. The same dataset-specific filters used for substitutions as described above were also applied to indels. Subsequently, indels were genotyped, requiring a VAF >0.2, a minimum depth of 10, and support from at least 5 mutant reads.

Structural variant and copy-number calling

Structural variants (SVs) were detected using GRIDSS[3], and confirmed visually to be adhering to the expected phylogenetic distribution based on single-base substitutions (SBS). SVs larger than 1kb with QUAL >=250 and those smaller than 30kb with QUAL >=300 were retained. Additionally, SVs required support from at least four discordant and two split reads, with a standard deviation of alignment positions > five being filtered out. A panel of normal samples (n=350) was incorporated to the GRIDSS panel to eliminate potential germline SVs and artefacts.

Autosomal copy number aberrations (CNAs) were identified using ASCAT (Allele-Specific Copy number Analysis of Tumours)[5]. The matched normal sample with coverage > 15X and no Y loss was selected for as for call structure variants. The in-house algorithm BRASS (Breakpoint AnalySiS)[4] was used to call CNAs on sex chromosomes by generating read count information across 500bp segments. Y loss was determined by comparing X and Y chromosome coverage means, validated through visual inspection of read depth.

Supplementary Figure 2: Validation of variant filtering approach

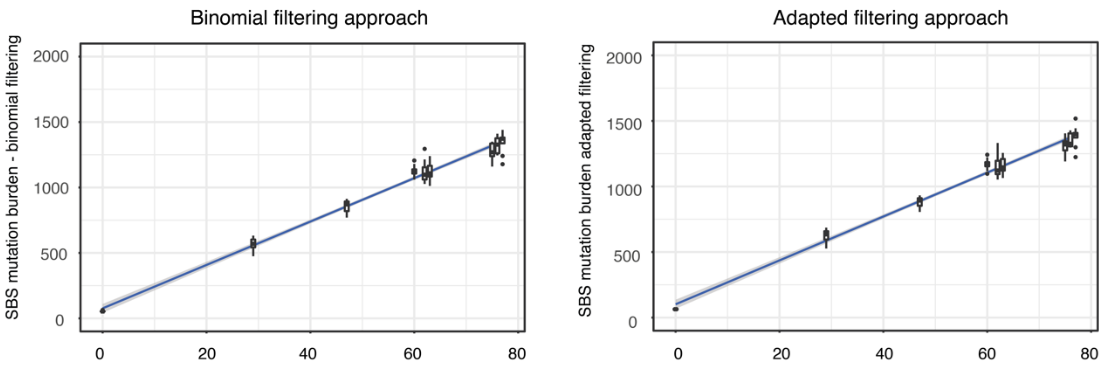

Supplementary Figure 2| Validation of variant filtering approach. a, SNV mutation burden of 10 samples per normal individual with variants filtered using the binomial filtering strategy that made use of several hundred other samples sequenced from the individual (left). SNV mutation burden of the same 10 samples per normal individual with variants filtered using the adapted filtering strategy that required only 10 samples per individual (right).

Construction of phylogenetic trees

MPBoot, a maximum parsimony tree approximation method[5], was used to build and annotate phylogenetic trees of the relationships between their sampled HSPCs as previously described[6].

Detailed information on the steps outlined in the main methods is provided below:

MPBoot, a maximum parsimony tree approximation method[7], was used to build phylogenetic trees of the relationships between the sampled cells. Variants were genotyped as 'present' (coded as 1) in a sample if 2 or more variant reads supported the variant. Variants were genotyped as 'absent' (coded as 0) in a sample if 0 variant reads were present at a given site and depth at that site was 6 or more. Sites that did not fall into either of the above categories were marked as 'unknown' (coded as 0.5). In all cases only a small minority of sites (< 5%) were categorised as 'unknown' or 'missing data' as shown in the table below.

The genotype matrix of shared variants was converted to a 'DNA string' for each sample with 'W' representing a 'wildtype' position, 'V' a 'variant' position and '?' representing 'unknown'. The DNA strings were then used as the input for *MPBoot*, which outputs unscaled trees with uninformative branch lengths. We explicitly added a 'dummy sample' (called 'ancestral') into the DNA strings that *MPBoot* used, which has non-mutant genotypes across all sites i.e. representing the genotypes of the reference genome. After tree construction the 'ancestral' branch was dropped prior to downstream analyses. A maximum likelihood approach and the original count data was then used to assign each mutation in an individual's dataset to a branch in their *MPBoot* generated phylogenetic tree (https://github.com/Nangalialab/treemut). Tree edge lengths were then made proportional to the number of mutations assigned to the branch.

The sensitivity of mutation calling in each sample was used to correct phylogeny branch lengths for sequencing coverage. Sensitivity was calculated as the fraction of known germline variants identified by CaVEMan in a specific sample. Mutation burden was corrected by multiplying the number of variants by 1/sensitivity for private branches. The sensitivity was adjusted to allow for the higher sensitivity on shared branches due to multiple samples containing the variant. Specifically, sensitivity was assessed by measuring the ability of the mutation-calling algorithms to detect heterozygous germline single nucleotide polymorphisms (SNPs) in each sample. Heterozygous SNPs should have the same VAF distribution and sensitivity as true somatic mutations. For private branches, the SNV component of branch lengths was scaled according to:

$$= \frac{n_{SNV}}{p_i}$$

Where n_{cSNV} is the corrected number of SNVs in sample i, n_{SNV} is the uncorrected number of SNVs called in sample i and p_i is the proportion of germline SNPs called by the Caveman algorithm in sample i.

For shared branches, it was assumed that (1) the regions of low sensitivity were independent between samples, (2) if a somatic mutation was called in at least one sample within the clade, it would also be correctly called (or 'rescued') in other samples in the clade (even in lower sensitivity samples). Shared branches were therefore scaled according to:

$$\frac{n_{SNV}}{1 - \pi_i(1 - p_i)}$$

Where the product is taken for $1 - p_i$ for each sample i within the clade. However, both of these assumptions will not hold true in all cases. Firstly, regions with low coverage are not randomly distributed, with some genomic regions likely to have low coverage in multiple samples. Secondly, while many mutations will be 'rescued' in subsequent samples once they have been called in a first sample - because the *treemut* algorithm for mutation assignment uses original read count data, meaning that even a single variant read in a subsequent sample is likely to result in the mutation being correctly assigned - this will not be true in every case. Some samples with very low coverage have 0 variant reads at a given site will by chance. In this situation, a mutation may not be correctly placed. While these factors may lead to an under-correction of shared branches, this approach provides a reasonable approximation. Corrected SNV burdens for each sample can then be calculated as the sum of corrected ancestral branch lengths back to the root of the phylogeny.

The phylogenies were then made ultrametric (or linearised) using a previously published bespoke algorithm to make all branch lengths equal[1]. Starting from the root of the tree and moving progressively towards each tip, the fraction of time for the given shared branch is calculated as the fraction of remaining time times the number of mutations on the given shared branch divided by the mean number of mutations of all descendants from that shared branch. The function is called recursively, updating the fraction of remaining time, as the algorithm moves from root to tip. This algorithm therefore has the property that the most confident timings (nodes near the root) are defined first, anchoring the timings of subsequent, less confident nodes.

Additional information in the form of driver mutations was then overlaid on the final ultrametric version of to generate the final phylogenies depicted in **Fig.5 and Extended Data Figs.8,9**.

To estimate the number of somatic mutations that may have already been acquired by PD37580 by age 13 (prior to commencing chlorambucil), we used the linear mixed model defined in Mitchell et al[1]. This model estimates an intercept of 54.57 (ie the mean number of somatic mutations present at birth), with a slope of 16.832 representing the mean number of somatic mutations acquired each year of life. This results in an expected mean somatic mutation burden of 273 at age 13. Assuming this mutation burden is Poisson distributed provides a 95% prediction interval of 241-306.

Analysis of driver variants

Variants identified were annotated with VAGrENT (Variation Annotation GENeraTor) (https://github.com/cancerit/VAGrENT) to identify protein coding mutations and putative driver mutations in each dataset. **Supplementary Table 9** lists the 18 genes we have used as our top clonal haematopoiesis genes (those identified by Fabre *et al* as being under positive selection in a targeted sequencing dataset of 385 older individuals, with CHEK2 added as being an additional gene commonly under positive selection in chemotherapy exposed individuals). 'Oncogenic' mutations (as assessed by EM) are shown in **Fig.5 and Extended Data Figs.8,9**.

Bulk cell sorts for Nanoseq sequencing (chemotherapy-exposed samples)

Chemotherapy-exposed samples:
Mononuclear cells were stained for 30 minutes at 4C in PBS/3%FCS containing the following antibodies: Zombie Aqua (1 in 400), CD3 APC (1 in 80), CD19 AF700 (1 in 80), CD45RA PerCPCy5.5 (1 in 80), CCR7 BV711 (1 in 80), CD14 BV605 (1 in 80). Cells were then washed and resuspended in PBS/3%FBS for cell sorting. Either a BD Aria III or BD Aria Fusion cell sorter (BD Biosciences) was used to sort various mature cell compartments (B cells, T naive cells, T memory cells, and monocytes) at the NIHR Cambridge BRC Cell Phenotyping hub. For each cell type ~40,000 cells were sorted into Eppendorf tubes containing 50 µl PBS. Further details in **Supplementary Table 10** and **Supplementary Fig.3.**

Supplementary figure 3: Flow sorting strategy for bulk cell sorts (chemotherapy exposed)

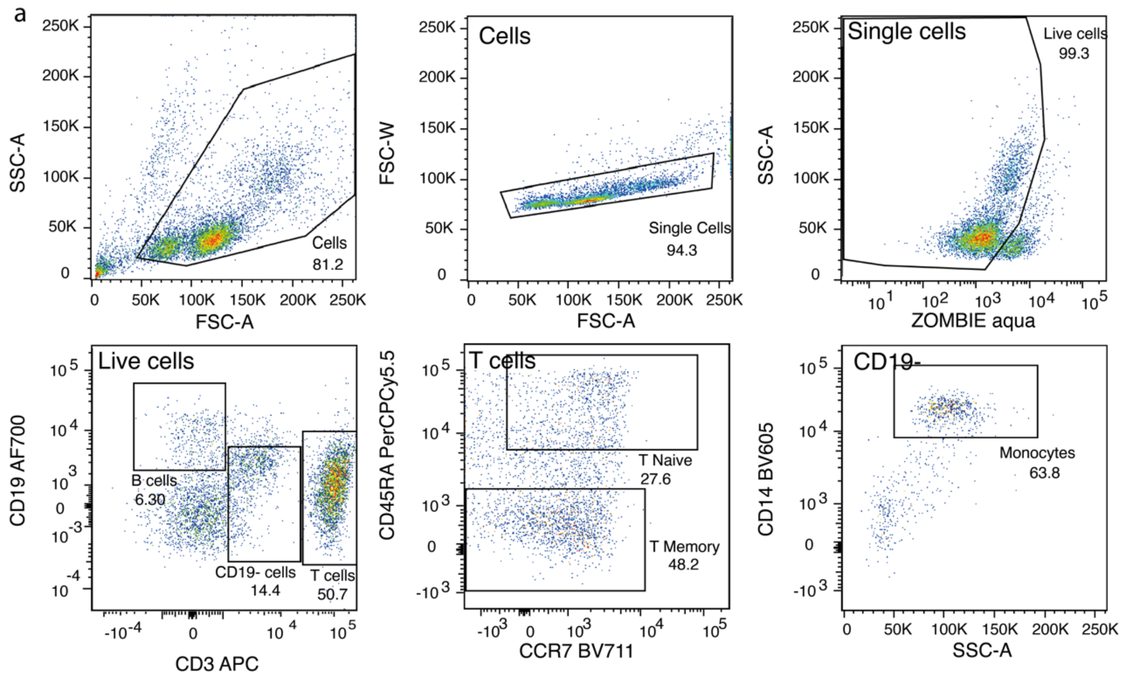

Supplementary Figure 3| Flow-sorting strategy for single mature blood cells from chemotherapy exposed individuals. a, Sorting of single human mature blood cells from peripheral blood. Cells were stained with the panel of antibodies in **Table S4** then single cells were bulk sorted according to the strategy depicted into individual Eppendorf tubes.

Unexposed normal samples:
Mononuclear cells were stained for 30 minutes at 4C in PBS/3%FCS containing the following antibodies: CD3 APC (1 in 80), CD4 BV785 (1 in 80), CD8 BV785 (1 in 40), CD14 BV605 (1 in 80), CD19 AF700 (1 in 80), CD20 PE Dazzle (1 in 80), CD27 BV421 (1 in 80), CD34 APC-Cy7 (1 in 27), CD38 FITC (1 in 80), CD45RA PerCPCy5.5 (1 in 80), CD56 PE (1 in 80), CCR7 BV711 (1 in 80), IgD PECy7 (1 in 100), Zombie Aqua (1 in 400). Cells were then washed and resuspended in PBS/3%FBS for cell sorting. Either a BD Aria III or BD Aria Fusion cell sorter (BD Biosciences) was used to sort various mature cell compartments (B cells, CD4+T naive cells, CD4+T memory cells, CD8+ T naïve cells, CD8+ T memory cells and monocytes) at the NIHR Cambridge BRC Cell Phenotyping hub. For each cell type ~40,000 cells were sorted into Eppendorf tubes containing 50 µl PBS. Further details in **Supplementary Table 11** and **Supplementary Fig.4**.

Supplementary Figure 4: Flow sorting strategy for bulk cell sorts (unexposed)

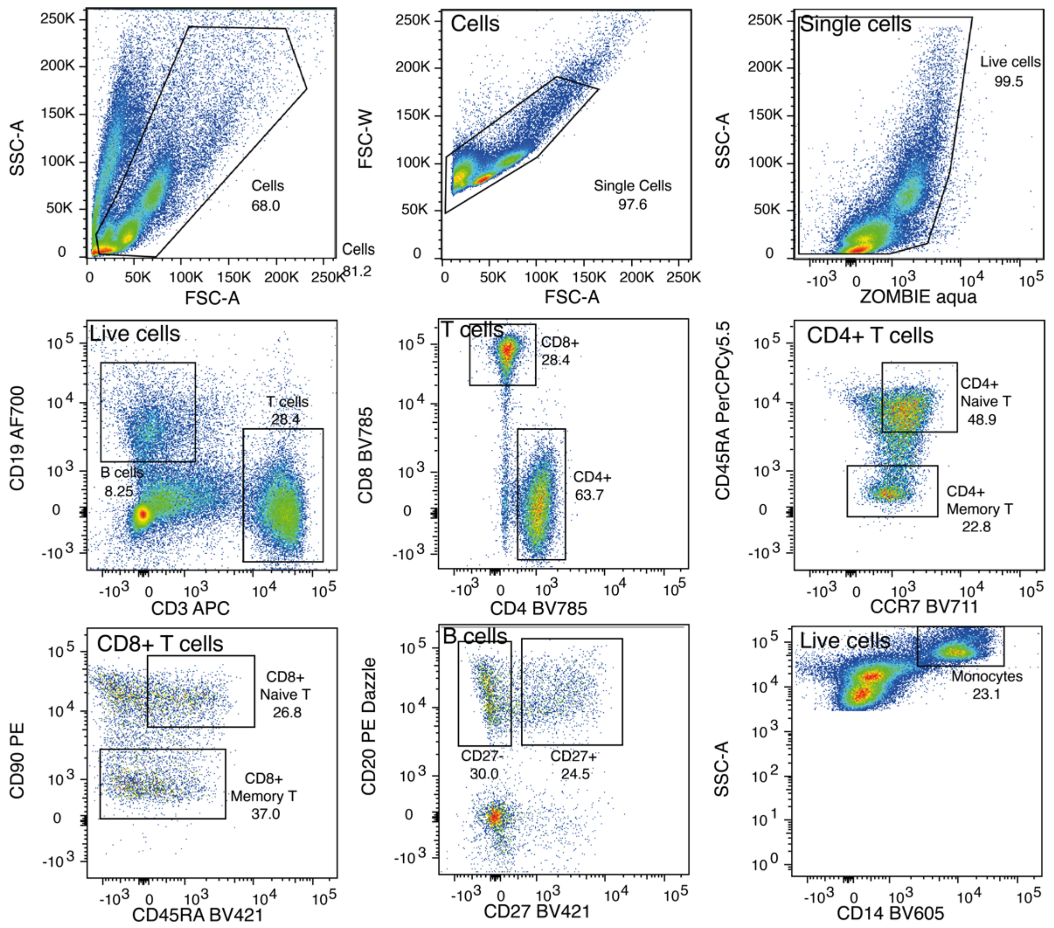

Supplementary Figure 4| Flow-sorting strategy for single mature blood cells from unexposed normal individuals. a, Sorting of single human mature blood cells from peripheral blood. Cells were stained with the panel of antibodies in **Table S5** then single cells were bulk sorted according to the strategy depicted into individual Eppendorf tubes.

Nanoseq (duplex) sequencing

Extracted DNA (1-5ng) from bulk cell sorts was submitted to the NanoSeq pipeline for library preparation and sequencing, as has been described in the previous paper[8]. They were then purified using 50µl water and 50µl Ampure XP beads (Beckman Coulter) at room temperature. After a 5-min binding reaction and magnetic bead separation, genomic DNA was washed twice with 75% ethanol, then eluted with 20µl nuclease-free water (NFW). Subsequently, 20 µl of the bead suspension was subjected to an on-bead fragmentation reaction. This fragmentation process took place in a final volume of 25 µl, containing 2.5 µl of 10× CutSmart buffer (consisting of 500 mM potassium acetate, 200 mM Tris-acetate, 100 mM magnesium acetate, 1 mg ml^{-1} BSA, pH 7.9 at 25 °C), 0.5 µl of 5 U $µl^{-1}$ HpyCH4V, and 2 µl NFW. The fragmentation reactions were then incubated at 37 °C for 15 min, followed by purification with 2.5× AMPure XP beads and resuspension in 15 µl NFW. Subsequent A-tailing of the fragmented DNA was conducted in 15 µl reactions, including 10 µl of fragmentation product, 1.5 µl of 10× NEBuffer 4 (consisting of 500 mM potassium acetate, 200 mM Tris-acetate, 100 mM magnesium acetate, 10 mM DTT, pH 7.9 at 25 °C), 0.15 µl of 5 U $µl^{-1}$ Klenow fragment (3′ to 5′ exo-, NEB), either 1.5 µl of 1 mM dATP or 1.5 µl of 1 mM equimolar dATP/ddBTPs , and 1.85 µl NFW. Here, ddBTPs refer to ddTTP, ddCTP, and ddGTP. The reactions were incubated at 37 °C for 30 min. Following this, the 15-µl A-tailing reaction product was combined with 22.4 µl of ligation mix, comprising 2.24 µl of 10× NEBuffer 4, 3.74 µl of 10 mM ATP, 0.33 µl of 15 µM xGen Duplex Seq Adapters (IDT, 1080799), 0.56 µl of 400 U $µl^{-1}$ T4 DNA ligase (NEB), and 15.53 µl NFW. The resulting reactions were incubated at 20 °C for 20 min and subsequently purified with 1× AMPure XP beads, followed by resuspension in 50 µl of NFW.

DNA was quantified by qPCR using a KAPA library quantification kit (KK4835). Samples were diluted in NFW to the standard amount (0.3 fmol for a 15X run) to reach a final volume of 25µl. Subsequently, libraries were amplified using PCR and cleaned up using two consecutive 0.7x AMPure XP beads.

We generated paired-end sequencing reads (150PE) using Illumina NovaSeq platform, resulting in a minimum of 20X per sample.

The sequencing data were then analysed with the BotSeq bioinformatics pipeline (v2.3.2), as described in the previous paper[8]. Sequences were aligned to the human reference genome (hs37d5) using BWA-MEM. Matched normal samples were used to filter out germline SNP from NanoSeq samples. We called indels and normalised the output using bcftools[9]. To assess the authenticity of DNA samples, we used VerifyBamID2[10], and removed all samples with a contamination level > 1% (3 from PD47541, 3 from PD50306, 2 from PD47695). The correction of mutation burden and trinucleotide substitution profiles was performed within the BotSeq pipeline.

HDP mutational signature analysis

Hierarchical Dirichlet process (HDP; https://github.com/nicolaroberts/hdp), based on the Bayesian hierarchical Dirichlet process, was used to extract mutational signatures. HDP was run without priors on SBS derived from phylogenetic trees obtained from HSPCs and mutations from NanoSeq samples. The NanoSeq mutations were corrected for the trinucleotide context abundance for each sample. This analysis was performed with a single hierarchy, enabling the sharing of mutational signatures information across different groups of data: samples generated from colony sequencing and those from Nanoseq sequencing methods. Both the clustering hyperparameters, alpha and beta, were set to one. The Gibbs sampler was run for 30,000 iterations (parameter “burnin”), with spacing of 200 iterations (parameter “space”) and

then 100 iterations (parameter “n”) were collected. After each iteration, three iterations of concentration parameters were performed. Twelve components were extracted, of which four components appeared to be combinations of previously reported signatures.

“Cosine similarity is used to compare the observed and the reference signatures, A and B, each characterised by K mutation types, as below:

$$\mathrm{sim}(A, B) = \frac{\sum_{k=1}^{K} A_k B_k}{\sqrt{\sum_{k=1}^{K} (A_k)^2}\sqrt{\sum_{k=1}^{K} (B_k)^2}}.$$

Given that the elements of A and B are non-negative, the cosine similarity values range from 0 to 1. A value of 1 implies the two signatures are identical, whereas a value of 0 means they are completely different. Therefore, the closer the cosine similarity is to 1 indicates the closer match between the extracted signature and the reference signature[11].

References

1. Lee-Six, H. *et al.* The landscape of somatic mutation in normal colorectal epithelial cells. *Nature* **574**, 532–537 (2019).
2. Van Loo, P. *et al.* Allele-specific copy number analysis of tumors. *Proceedings of the National Academy of Sciences of the United States of America* **107**, 16910–16915 (2010).
3. Cameron, D. L. *et al.* GRIDSS: sensitive and specific genomic rearrangement detection using positional de Bruijn graph assembly. (2017) doi:10.1101/gr.222109.117.
4. Nik-Zainal, S. *et al.* The Life History of 21 Breast Cancers. *Cell* **149**, 994–1007 (2012).
5. Thi Hoang, D. *et al.* MPBoot: fast phylogenetic maximum parsimony tree inference and bootstrap approximation. doi:10.1186/s12862-018-1131-3.
6. Mitchell, E. *et al.* Clonal dynamics of haematopoiesis across the human lifespan. *Nature* **606**, 343–350 (2022).
7. Fabre, M. A. *et al.* The longitudinal dynamics and natural history of clonal haematopoiesis. *Nature* **606**, 335–342 (2022).
8. Abascal, F. *et al.* Somatic mutation landscapes at single-molecule resolution. *Nature* **593**, 405–410 (2021).
9. Danecek, P. *et al.* Twelve years of SAMtools and BCFtools. *GigaScience* **10**, giab008 (2021).
10. Zhang, F. *et al.* Ancestry-agnostic estimation of DNA sample contamination from sequence reads. *Genome Res* **30**, 185–194 (2020).
11. Alexandrov, L. B., Nik-Zainal, S., Wedge, D. C., Campbell, P. J. & Stratton, M. R. Deciphering Signatures of Mutational Processes Operative in Human Cancer. *Cell Reports* **3**, 246–259 (2013).
