# Supplementary material for: The long-term effects of chemotherapy on normal blood cells
Source: Nat Genet. 2025 Jul 1;57(7):1684–94. doi: 10.1038/s41588-025-02234-x (PMC12283364; doi:10.1038/s41588-025-02234-x)
Supplement: Supplementary file 2 — Reporting Summary [file 41588_2025_2234_MOESM2_ESM.pdf]

nature portfolio

Corresponding author(s): Michael Stratton

Last updated by author(s): Feb 12, 2025

Reporting Summary

Nature Portfolio wishes to improve the reproducibility of the work that we publish. This form provides structure for consistency and transparency in reporting. For further information on Nature Portfolio policies, see our Editorial Policies and the Editorial Policy Checklist.

Statistics

For all statistical analyses, confirm that the following items are present in the figure legend, table legend, main text, or Methods section.

n/a	Confirmed	
☐	☒	The exact sample size (*n*) for each experimental group/condition, given as a discrete number and unit of measurement
☐	☒	A statement on whether measurements were taken from distinct samples or whether the same sample was measured repeatedly
☐	☒	The statistical test(s) used AND whether they are one- or two-sided<br>*Only common tests should be described solely by name; describe more complex techniques in the Methods section.*
☐	☒	A description of all covariates tested
☐	☒	A description of any assumptions or corrections, such as tests of normality and adjustment for multiple comparisons
☐	☒	A full description of the statistical parameters including central tendency (e.g. means) or other basic estimates (e.g. regression coefficient) AND variation (e.g. standard deviation) or associated estimates of uncertainty (e.g. confidence intervals)
☐	☒	For null hypothesis testing, the test statistic (e.g. *F*, *t*, *r*) with confidence intervals, effect sizes, degrees of freedom and *P* value noted<br>*Give P values as exact values whenever suitable.*
☒	☐	For Bayesian analysis, information on the choice of priors and Markov chain Monte Carlo settings
☒	☐	For hierarchical and complex designs, identification of the appropriate level for tests and full reporting of outcomes
☒	☐	Estimates of effect sizes (e.g. Cohen's *d*, Pearson's *r*), indicating how they were calculated

Our web collection on statistics for biologists contains articles on many of the points above.

Software and code

Policy information about availability of computer code

Data collection	None.
Data analysis	Open source programs used (also stated in manuscript):<br>List of programs and softwares:<br>• R: version 3.6.1<br>• BWA-MEM: version 0.7.17/0.7.5a-r405 (https://sourceforge.net/projects/bio-bwa/)<br>• cgpCaVEMan: version 1.11.2/1.13.14/1.14.1 (https://github.com/cancerit/CaVEMan)<br>• cgpPindel: version 2.2.5/3.2.0/3.3.0 (https://github.com/cancerit/cgpPindel)<br>• Brass: version 6.1.2/6.2.0/6.3.0/6.3.4 (https://github.com/cancerit/BRASS)<br>• ASCAT NGS: version 4.2.1/4.3.3 (https://github.com/cancerit/ascatNgs)<br>• VAGrENT: version 3.5.2/3.6.0/3.6.1 (https://github.com/cancerit/VAGrENT)<br>• GRIDSS: version 2.9.4 (https://github.com/PapenfussLab/gridss)<br>• MPBoot: version 1.1.0 (https://github.com/diepthihoang/mpboot)<br>• cgpVAF: version 2.4.0 (https://github.com/cancerit/vafCorrect)<br>• FlowJo: version 10<br>• HDP: (https://github.com/nicolaroberts/hdp)<br>• bcftools: version 1.18 (https://github.com/samtools/bcftools)<br>• VerifyBamID2: version 2.0.1 (https://anaconda.org/bioconda/verifybamid2)<br>• mSigHDP: version 2.1.2 (https://github.com/steverozen/mSigHdp)<br>• SigProfilerAssignment: version 0.1.0 (https://github.com/AlexandrovLab/SigProfilerAssignment)

Custom code made available (also stated in manuscript): https://github.com/emily-mitchell/chemotherapy
No commercial software used.

For manuscripts utilizing custom algorithms or software that are central to the research but not yet described in published literature, software must be made available to editors and reviewers. We strongly encourage code deposition in a community repository (e.g. GitHub). See the Nature Portfolio guidelines for submitting code & software for further information.

Data

Policy information about availability of data

All manuscripts must include a data availability statement. This statement should provide the following information, where applicable:

- Accession codes, unique identifiers, or web links for publicly available datasets
- A description of any restrictions on data availability
- For clinical datasets or third party data, please ensure that the statement adheres to our policy

Sequence data that support the findings of this study have been deposited in the European Genome-Phenome Archive (https://www.ebi.ac.uk/ega/home). Additional data is available on github (https://github.com/emily-mitchell/chemotherapy/). Raw sequencing data is available on EGA (accession number WGS dataset EGAD00001015339 and Nanoseq dataset EGAD00001015340). The main data needed to reanalyse / reproduce the results presented is available on Mendeley Data (DOI: 10.17632/2fczcd49yj.1).

Publically available datasets used:
Human reference genome (NCBI build37)

Research involving human participants, their data, or biological material

Policy information about studies with human participants or human data. See also policy information about sex, gender (identity/presentation), and sexual orientation and race, ethnicity and racism.

Reporting on sex and gender	Information on age and sex is included for all individuals in Supplementary table 1 and Fig. 1a.
Reporting on race, ethnicity, or other socially relevant groupings	Information on race and ethnicity have not been reported / provided in this study.
Population characteristics	All relevant information about donors is provided in Supplementary table 1, which includes information on age, sex, diagnoses, treatment regimens, time since exposure to chemotherapy, any exposure to radiotherapy. The number of cycles of each chemotherapy is also provided.
Recruitment	22 chemotherapy exposed participants were recruited from oncology / haematology clinics at Addenbrooke's Hospital Cambridge, with the only inclusion criteria being that they had been exposed to chemotherapy. One chemotherapy exposed participant was recruited from MD Anderson Cancer Centre. 7 unexposed normal individuals had been recruited to a previously published study (Mitchell et al 2022). Two additional normal donors were recruited from Cambridge Biorepository for Translational Medicine with no specific inclusion criteria other than that they had not been previously exposed to chemotherapy.
Ethics oversight	Blood or bone marrow samples from individuals un-exposed to chemotherapy were obtained from three sources: 1) Stem Cell Technologies provided frozen mononuclear cells (MNCs) for the cord blood sample that had been collected with informed consent, including for whole genome sequencing (catalog #70007); all data previously published. 2) Cambridge Blood and Stem Cell Biobank (CBSB) provided fresh peripheral blood samples taken with informed consent from two patients at Addenbrooke’s Hospital (NHS Cambridgeshire 4 Research Ethics Committee reference 07/MRE05/44 for samples collected pre-November 2019 and Cambridge East Ethics Committee reference 18/EE/0199 for samples collected from November 2019 onwards; all data previously published. 3) Cambridge Biorepository for Translational Medicine (CBTM) provided frozen bone marrow +/- peripheral blood MNCs taken with informed consent from seven deceased organ donors. Samples were collected at the time of abdominal organ harvest (Cambridgeshire 4 Research Ethics Committee reference 15/EE/0152); data previously published from 4 individuals with new data generated from an additional 2 individuals (PD49236 and PD49327).<br><br>Blood samples from individuals previously exposed to chemotherapy were obtained from two sources: 1) Cambridge Blood and Stem Cell Biobank (CBSB) provided fresh peripheral blood samples taken with informed consent from 22 patients at Addenbrooke’s Hospital (NHS Cambridgeshire 4 Research Ethics Committee reference 07/MRE05/44 for samples collected pre-November 2019 and Cambridge East Ethics Committee reference 18/EE/0199 for samples collected from November 2019 onwards; all unpublished data. One chemotherapy exposed individual, PD44703, had two samples taken at timepoints a year apart. All others were sampled at a single timepoint. 2) Baylor College of Medicine provided single cell-derived haematopoietic colonies from bone marrow taken following informed consent from 1 patient from MD Anderson Cancer Centre; Research Ethics Committee of the University of Texas MD Anderson Cancer Centre Institutional Review Board reference PA12-0305 (genomic analysis protocol) and LAB01-473 (laboratory protocol).

Note that full information on the approval of the study protocol must also be provided in the manuscript.

Field-specific reporting

Please select the one below that is the best fit for your research. If you are not sure, read the appropriate sections before making your selection.

☒ Life sciences ☐ Behavioural & social sciences ☐ Ecological, evolutionary & environmental sciences

For a reference copy of the document with all sections, see nature.com/documents/nr-reporting-summary-flat.pdf

Life sciences study design

All studies must disclose on these points even when the disclosure is negative.

Sample size	We optimised the number of chemotherapy exposed individuals (23) and number of haematopoietic stem cells sequenced at higher depth per individual (4-10) to describe the mutation burden and mutational signatures in haematopoietic stem and progenitor cells across a range of chemotherapy exposures. Duplex sequencing was used to allow interrogation of the same information in mature blood cell subsets for 18 chemotherapy exposed individuals. In addition for a subset of six chemotherapy exposed individuals and five normal individuals, we sequenced larger numbers of HSPC colonies to provide a larger dataset for mutational signature analysis and describe changes in clonal structure with chemotherapy exposure. No power calculation was performed, and there was no target effect size. No sample size calculation was performed.
Data exclusions	Per pre-established criteria, genomes with a sequencing depth of less than 7X (23 samples) or with a VAF distribution showing evidence of non-clonality or contamination (peak VAF < 40%) (29 samples) were excluded from the analysis.
Replication	While the specific donor samples used have been exhausted, the results from this study should be generally reproducible in separate individuals of the same age and chemotherapy exposures, using the protocols and code included in this manuscript. For a single individual the haematopoietic stem and progenitor cell phylogeny was reconstructed from samples taken at two timepoints one year apart and show reproducible results. In some ways each individual exposed to an chemotherapeutic agent represents a an experimental replication: oxaliplatin n=8; carboplatin n = 2; cyclophosphamide n = 8; bendamustine n = 5; chlorambucil n = 2; 5FU/capecitabine n = 9; irinotecan n=5; doxorubicin n = 4; etoposide n = 4; vincristine n = 7. All other agents included were only received by a single individual.
Randomization	This is not relevant to our study. All individuals were haematopoietically normal, and there was no test versus control groups.
Blinding	Blinding was not relevant to our study. There was no test performed that required blinding.

Reporting for specific materials, systems and methods

We require information from authors about some types of materials, experimental systems and methods used in many studies. Here, indicate whether each material, system or method listed is relevant to your study. If you are not sure if a list item applies to your research, read the appropriate section before selecting a response.

Materials & experimental systems

n/a	Involved in the study	
☐	☒	Antibodies
☒	☐	Eukaryotic cell lines
☒	☐	Palaeontology and archaeology
☒	☐	Animals and other organisms
☒	☐	Clinical data
☒	☐	Dual use research of concern
☒	☐	Plants

Methods

n/a	Involved in the study	
☒	☐	ChIP-seq
☒	☐	Flow cytometry
☒	☐	MRI-based neuroimaging

Antibodies

Antibodies used

Marker; Fluorochrome; Manufacter; Catalogue Number; Clone; Dilution; Citation
CD3; FITC; BD; 555339; HIT3a; 1 in 500; Beverley PC et al. Eur J Immunol. 1981; 11(4):329-334.
CD90; PE; Biolgend; 328110; 5E10; 1 in 50; Adutler-Lieber S, et al. 2013. J Cardiovasc Pharmacol Therap. 18:78.
CD49f; PECy5; BD; 551129; GoH3; 1 in 100; Aumailley et al. Exp Cell Res. 1990; 188(1):55-60.
CD19; A700; Biolgend; 302226; HIB19; 1 in 300; Boyle M, et al. 2015. J Infect Dis. 212: 416-425.
CD34; APCCy7; Biolgend; 343514; 581; 1 in 100; Bigley V, et al. 2011. J Exp Med. 208:227.
Zombie ; Aqua; Biolgend; 423101; NA; 1 in 2000; Berg J, et al. 2013. J Exp Med. 210:2803.
CD38; PECy7; Biolgend; 303516; HIT2; 1 in 100; Chaimowitz N, et al. 2011. J Immunol. 187:5114.
CD45RA; BV421; Biolgend; 304130; HI100; 1 in 100; Causi E, et al. 2015. PLoS One. 10: 0136717.

Marker; Fluorochrome; Manufacter; Catalogue Number; Clone; Dilution; Citation
Zombie; Aqua; biolegend; 423101; NA; 1 in 400; Berg J, et al. 2013. J Exp Med. 210:2803.

CD3; APC; biolegend; 300301; HIT3A; 1 in 80; Kaushal A, et al. 2021. Blood Cancer Discov. 2:600
CD4; BV785; biolegend; 317401; OKT4; 1 in 80; Jung IY, et al. 2022. Sci Transl Med. 14:eabn7336.
CD8 ; BV785; biolegend; 301045; RPA-T8; 1 in 40; Swadling L, et al. 2020. Cell Rep. 30:687.
CD14; BV605; biolegend; 367125; 63D3; 1 in 80; Perry JSA, et al. 2018. Immunity. 48:923.
CD19; AF700; biolegend; 302225; HIB19; 1 in 80; Viny AD, et al. 2019. Cell Stem Cell. 25:682
CD20; PE Dazzle; biolegend; 302347; 2H7; 1 in 80; Brouwer PJM, et al. 2020. Science. 369:643
CD27; BV421; biolegend; 356429; M-T271; 1 in 80; Tran TM, et al. 2020. Immunity. 51(4):750-765.
CD34; Apc-Cy7; biolegend; 343513; 581; 1 in 27; Takayama N, et al. 2020. Cell Stem Cell. 28(3):488-501.e10
CD38; FITC; biolegend; 980304; HIT2; 1 in 80; NA
CD45RA; PerCPCy5.5; biolegend; 304121; HI100; 1 in 80; Sammicheli S, et al. 2012. J Autoimmun. 38:304
CD56; PE; biolegend; 355503; 39D5; 1 in 80; de Andrade LF, et al. 2019. JCI Insight. 4:e133103
CCR7; BV711; biolegend; 353227; G043H7; 1 in 80; Arunachalam PS, et al. 2021. Nature. 596:410.
IgD; PECy7; biolegend; 348209; IA6-2; 1 in 100; Rouers A, et al. 2021. Cell Rep Med. 2:100278

Marker; Fluorochrome; Manufacter; Catalogue Number; Clone; Dilution; Citation
Zombie; Aqua; biolegend; 423101; NA; 1 in 400; Berg J, et al. 2013. J Exp Med. 210:2803.
CD3 ; APC; biolegend; 300301; HIT3A; 1 in 80; Kaushal A, et al. 2021. Blood Cancer Discov. 2:600
CD19; AF700; biolegend; 302225; HIB19; 1 in 80; Viny AD, et al. 2019. Cell Stem Cell. 25:682
CD45RA ; PerCPCy5.5; biolegend; 304121; HI100; 1 in 80; Sammicheli S, et al. 2012. J Autoimmun. 38:304
CCR7; BV711; biolegend; 353227; G043H7; 1 in 80; Arunachalam PS, et al. 2021. Nature. 596:410.
CD14; BV605; biolegend; 367125; 63D3; 1 in 80; Perry JSA, et al. 2018. Immunity. 48:923.

Validation

These werre all previously used and validated antibodies.

Plants

Seed stocks

NA

Novel plant genotypes

NA

Authentication

NA
